# REACH-ASD: a UK randomised controlled trial of a new post-diagnostic psycho-education and acceptance and commitment therapy programme against treatment-as-usual for improving the mental health and adjustment of caregivers of children recently diagnosed with autism spectrum disorder

**DOI:** 10.1186/s13063-022-06524-1

**Published:** 2022-07-22

**Authors:** Kathy Leadbitter, Richard Smallman, Kirsty James, Gemma Shields, Ceri Ellis, Sophie Langhorne, Louisa Harrison, Latha Hackett, Alison Dunkerley, Leo Kroll, Linda Davies, Richard Emsley, Penny Bee, Jonathan Green, The REACH-ASD Team, Sofia Ahmed, Hilary Beach, Charlotte Butter, June Gilbert, Caitlin Goldie, Rebekah Howell, Tessa Hutton, Amelia Pearson, Katy Roe, Cameron Sawyer, Amy Van Gils

**Affiliations:** 1 Division of Neuroscience and Experimental Psychology, University of Manchester, Manchester, UK; 2 Manchester University NHS Foundation Trust, Manchester, UK; 3 Department of Biostatistics and Health Informatics, Institute of Psychiatry, Psychology and Neuroscience, Kings College London, London, UK; 4 Pennine Care NHS Foundation Trust, Ashton-under-Lyne, UK

**Keywords:** Autism, Randomised controlled trial, Caregivers, Acceptance and commitment therapy, Psycho-education, Diagnosis

## Abstract

**Background:**

Autism is a neurodevelopmental disability affecting over 1% of UK children. The period following a child’s autism diagnosis can present real challenges in adaptation for families. Twenty to 50% of caregivers show clinically significant levels of mental health need within the post-diagnostic period and on an ongoing basis. Best practice guidelines recommend timely post-diagnostic family support. Current provision is patchy, largely unevidenced, and a source of dissatisfaction for both families and professionals. There is a pressing need for an evidenced programme of post-diagnostic support focusing on caregiver mental health and adjustment, alongside autism psycho-education. This trial tests the clinical and cost-effectiveness of a new brief manualised psychosocial intervention designed to address this gap.

**Methods:**

This is a multi-centre two-parallel-group single (researcher)-blinded randomised controlled trial of the Empower-Autism programme plus treatment-as-usual versus usual local post-diagnostic offer plus treatment-as-usual. Caregivers of children aged 2–15 years with a recent autism diagnosis will be recruited from North West England NHS or local authority centres. Randomisation is individually by child, with one “index” caregiver per child, stratified by centre, using 2:1 randomisation ratio to assist recruitment and timely intervention. Empower-Autism is a group-based, manualised, post-diagnostic programme that combines autism psycho-education and psychotherapeutic components based on Acceptance and Commitment Therapy to support caregiver mental health, stress management and adjustment to their child’s diagnosis. The comparator is any usual local group-based post-diagnostic psycho-education offer. Receipt of services will be specified through health economic data. Primary outcome: caregiver mental health (General Health Questionnaire-30) at 52-week follow-up. Secondary outcomes: key caregiver measures (wellbeing, self-efficacy, adjustment, autism knowledge) at 12-, 26- and 52-week follow-up and family and child outcomes (wellbeing and functioning) at 52-week endpoint. Sample: *N*=380 (approximately 253 intervention/127 treatment-as-usual). Primary analysis will follow intention-to-treat principles using linear mixed models with random intercepts for group membership and repeated measures. Cost-effectiveness acceptability analyses will be over 52 weeks, with decision modelling to extrapolate to longer time periods.

**Discussion:**

If effective, this new approach will fill a key gap in the provision of evidence-based care pathways for autistic children and their families.

**Trial registration:**

ISRCTN 45412843. Prospectively registered on 11 September 2019.

**Supplementary Information:**

The online version contains supplementary material available at 10.1186/s13063-022-06524-1.

## Administrative information

Note: the numbers in curly brackets in this protocol refer to SPIRIT checklist item numbers. The order of the items has been modified to group similar items (see http://www.equator-network.org/reporting-guidelines/spirit-2013-statement-defining-standard-protocol-items-for-clinical-trials/).

**Table:** 

Title {1}	REACH-ASD: A UK randomised controlled trial of a new post-diagnostic psycho-education and acceptance and commitment therapy programme against treatment-as-usual for improving the mental health and adjustment of caregivers of children recently diagnosed with Autism Spectrum Disorder
Trial registration {2a and 2b}.	ISRCTN ID:45412843. https://www.isrctn.com/ISRCTNISRCTN45412843. Prospectively registered on 11 September 2019
Protocol version {3}	24.02.22; Version 8
Funding {4}	UK National Institute for Health and Care Research Health Technology Assessment Board; Ref: 17/80/09 UK Department of Health Excess Treatment Costs subvention
Author details {5a}	Division of Neuroscience and Experimental Psychology, University of Manchester, UK Manchester University NHS Foundation Trust, UK Department of Biostatistics and Health Informatics, Institute of Psychiatry, Psychology and Neuroscience, Kings College London, UK Pennine Care NHS Foundation Trust, UK
Name and contact information for the trial sponsor {5b}	Manchester University NHS Foundation Trust, Dr. Lynne Webster, Central Research Office, Nowgen Building, Grafton Street, Manchester, M13 9WL, UK. Telephone 0044 161 2764125
Role of sponsor {5c}	The study sponsor and funders played no role in: the study design; the collection, management, analysis and interpretation of data; the writing of the report; and the decision to submit for publication.

## Introduction

### Background and rationale {6a}

#### REACH-ASD Trial

The trial tests the clinical and cost-effectiveness of a new manualised psychosocial intervention called Empower-Autism, designed to be deliverable within the NHS and to directly address the combined informational, relational, and emotional needs of caregivers in the post-diagnostic period and thereby improve the mental health and adjustment of caregivers of recently diagnosed autistic children.

#### Caregiver response to autism diagnosis

Autism spectrum disorder (autism) is a neurodevelopmental disability affecting over 1% of UK children, defined by differences in social reciprocity, communication, sensory processing, and patterns of behaviour, interests, and cognition. The impact of these clinical features is variable across individuals, but often bears a significant influence on development, functioning, and wellbeing across the lifespan. The period following a child’s diagnosis can present real challenges in adaptation for families. Twenty to 50% of caregivers show clinically significant levels of mental health need within the post-diagnostic period and on an ongoing basis [1, 2]. A recent systematic review of UK caregiver experiences of autism diagnosis in their child emphasised three distinct areas of post-diagnostic need: emotional, relational, and informational [3]. Emotional responses to the diagnosis are heterogeneous: feelings of grief, disorientation, and disempowerment are common; feelings of relief and validation also occur [4–6]. Caregivers of autistic children can experience social isolation, judgement, and stigma. Many actively want to understand and parent their child as best they can and actively seek out support with this. The mental health and wellbeing of caregivers is of public health importance in and of itself. In addition, improved caregiver wellbeing is likely to have downstream effects on family and child wellbeing and may result in more effective uptake of subsequent evidenced-based caregiver-mediated interventions known to bring long-term benefits for both the caregiver and their autistic child [7].

#### Current provision

Best practice guidelines [8–10] recommend provision of timely post-diagnostic family support. However, current provision is patchy across the UK and represents a source of increasing dissatisfaction for both families and professionals [11–13]. There has been a recent step change in identifying effective episodic interventions for autistic children within robust randomised trials. For example, current evidence supports the effectiveness of caregiver-mediated communication intervention strategies on dyadic caregiver-child interaction and on child autism impairments [14, 15]. Despite these significant developments and the fact that caregiver psycho-education is amongst the most used autism interventions, post-diagnostic psycho-social intervention for caregivers has received relatively little research attention and remains a large evidence gap, not only in the UK but internationally. There are long-standing local and national clinical initiatives to provide group-based caregiver psycho-education groups to address informational (and to some extent, relational) needs, some of which have evidence of acceptability or observational evidence suggestive of positive outcomes [16–19]. Internationally, there have been a small number of randomised controlled trials (RCTs) of psycho-education with generic/child outcomes [20, 21] and one RCT with a treatment effect on parental mental health [22]. However, there is no well powered trial to date and there remains a pressing need for an evidenced programme of post-diagnostic support for caregivers which includes a focus on caregiver emotional and mental health, as well as autism psycho-education.

#### Acceptance and commitment therapy

There is increasing focus within the autism context on interventions that support caregivers’ emotional needs, including adjustment to the diagnosis, long-term stress management, resilience, and stigma protection, with approaches such as mindfulness, cognitive restructuring, and Acceptance and Commitment Therapy (ACT; [23–25]). ACT [26] has a growing evidence base for effectiveness in adult mental health [27, 28]. It shares lineage with cognitive-behavioural interventions and shows similar general effectiveness [27]. Several teams have now recognised the relevance of ACT to the distinct psychological task faced by caregivers of newly diagnosed children due to the following: (i) its emphasis on psychological acceptance (validating challenging emotions and cognitions, rather than seeking to change them) [29]; (ii) incorporation of mindfulness techniques (successful in reducing caregiver and child mental health difficulties) [30, 31] but in a way that is more sustainable than full mindfulness interventions that have high time and training costs [32]; and (iii) a ‘core values’ focus that may help caregivers re-assert parenting values challenged by realisation of the child’s condition [32, 33]. A recent systematic review [34] identifies 8 studies investigating the use of ACT to improve the mental health of caregivers of autistic children: one small-scale randomised controlled trial, one quasi-experimental study, and six observational studies. The RCT (*N*=18; [35]) compared a four-hour ACT programme against no intervention and reported a large treatment effect on parental depression. Juvin and colleagues [34] concluded that there was preliminary promising evidence that ACT can be helpful for the parents of autistic children but that larger randomised controlled trials were needed. A second systematic review [36] focussing on neurodevelopmental disabilities (NDDs) more broadly found nine articles centred on autism and two on other NDDs and also concluded that there was provisional support for the use of ACT with caregivers of children with NDDs but that further research was needed. A more recent small randomised controlled trial (*N*=20; [37]), not included in the two systematic reviews, tested a 36-h ACT intervention for parents of autistic children against a more traditional parent training programme and reported significant reductions in parent stress in the ACT intervention group. These authors also pointed to the need for larger randomised controlled trials.

#### Development of Empower-Autism

Empower-Autism was designed during a pre-trial development and feasibility testing phase. It grew out of two pre-existing foundational approaches. The first was a psycho-educational workshop model developed and delivered over 10 years within the Manchester University NHS Foundation Trust Child and Adolescent Mental Health Service. Published evaluations of this approach reported excellent feasibility and acceptability [18, 19]. The second foundational approach was a manualised 5-h acceptance and commitment therapy programme developed specifically for caregivers of children with disabilities [38, 39], used with the developers’ consent. An RCT of this programme as part of a package for parents of children with acquired brain injury compared to treatment-as-usual (*N*=59; [38]) found moderate effects on parental stress, anxiety, and parenting confidence. In unpublished work, the same ACT programme then showed good applicability and acceptability when applied post-diagnostically with groups of parents of autistic children, with very positive qualitative feedback (Sofranoff, pers comm). A comprehensive and iterative stakeholder co-production process was undertaken to further develop and blend these two approaches, with additional elements as indicated by stakeholder involvement, informed by a Theory of Change framework [40]. The pre-trial feasibility phase provided quantitative and qualitative evidence of feasibility and acceptability of the new programme and recommendations for further refinement and co-design prior to finalising the intervention protocol for the RCT.

#### Response to COVID-19 pandemic

Onset of the COVID-19 pandemic coincided with pre-trial feasibility study of the in-person manual. In response, the trial team investigated online delivery of the programme through video-conferencing. The implications of a move to online delivery were discussed with stakeholders and optional online adaptations were included within the intervention manual. Following work to adapt the intervention manual for online delivery, an additional feasibility test of this adaptation was undertaken. This provided quantitative and qualitative evidence of the feasibility and acceptability of online delivery in relation to both practitioners and participants.

#### Empower-Autism

The final programme is described in detail below. In brief, it is a 15-h group-based programme, with associated online and physical resources, that integrates problem-focused psycho-education to enable empowerment through knowledge of autism, connection with peers, and the ability to make informed and positive choices for ongoing care, and ACT-informed psychotherapeutic components to support with the immediate challenge caregivers face in adjusting to the diagnosis and coping with the ongoing stress faced by many caregivers of disabled children. If shown to be effective, this theoretically based targeted approach to caregiver education, empowerment, and stress reduction will fill a key evidential gap in the provision of efficient and effective developmentally sequenced autism interventions from diagnosis onwards.

## Objectives {7}

### Overall aim

To evaluate the effectiveness and cost-effectiveness of the Empower-Autism intervention compared to treatment-as-usual

### Objective 1

To test the effectiveness of the Empower-Autism intervention over usual care on: (i) caregiver mental health (primary outcome); (ii) caregiver knowledge, wellbeing, health status, and adjustment; and (iii) parenting stress and self-efficacy, at 12-, 26- and 52-week follow-up

### Objective 2

To test the effect of the intervention on: (i) family wellbeing and (ii) child wellbeing, behaviour, and adaptive functioning at 52-week endpoint

### Objective 3

To assess (i) the net costs and quality-adjusted life years (QALYs) of the intervention compared to treatment-as-usual (TAU) and (ii) whether, when compared to TAU, the intervention is cost-effective from the perspective of NHS and social care

### Objective 4

To identify perceptions of the intervention and barriers to implementation within routine service provision (process evaluation)

## Trial design {8}

A multi-centre two parallel group single (researcher)-blinded randomised controlled trial of the Empower-Autism programme plus TAU versus the usual local post-diagnostic offer plus TAU. Caregivers in the trial intervention arm will access the Empower-Autism programme in place of their usual local post-diagnostic offer. Participants in the TAU arm will receive the usual post-diagnostic offer of their local area. Participants in both trial arms can access all other services and interventions on offer in their locality, as per usual care.

### Population

Parents/primary caregivers of children and young people aged 2–15 years with a recent autism diagnosis.

Individual randomisation by child, with one “index” caregiver per child, and stratification by centre, using 2:1 randomisation ratio to assist recruitment, allow for efficient group formation, reduce risk of drop-out between consent and intervention, and deliver timely post-diagnostic intervention. This is a partially nested design as there is group-level clustering in the intervention arm and no clustering in the control arm; the optimal procedure for such a design is for a greater number of participants allocated to the intervention arm to account for the intra-cluster correlations in the groups [41].

A 4-month internal pilot, using the fully developed intervention package and a full assessment battery, to test randomised design and recruitment within 4 study centres, with pre-specified progression criteria adopting a traffic light approach (target recruitment rate=60, 15/centre; green: ≥15 participants randomised in 3/4 centres; amber: 15 participants randomised in 2 centres; red: 15 participants randomised in <2 centres).

### Primary outcome

Caregiver mental health (General Health Questionnaire-30) at 52-week follow-up.

### Secondary outcomes

Key caregiver measures at 12-, 26-, and 52-week follow-up and family and child outcomes at 52-week endpoint. Sample: *N*=380 (approximately 253 intervention/127 TAU).

### Health economic evaluation

An economic evaluation integrated within the trial, using service use and health status data collected from participants, will investigate the cost-effectiveness of the Empower-Autism programme from the perspective of NHS and social care. Decision modelling will explore the potential cost-effectiveness of the intervention over longer time horizons.

### Nested qualitative process evaluation

This will draw on recognised theoretical frameworks to analyse intervention acceptability (Theoretical Framework of Acceptability; [42]) and inform intervention development, implementation, and sustainability (Normalisation Process Theory; [43]). Data will be collected post-endpoint from selected participants from both trial arms (*n*=15), as well as those disengaging from the intervention (*n*=5). Purposive sampling will ensure data is representative of the wider demographic. Semi-structured interviews will elicit descriptions of how participants have perceived and understood the intervention and how it has or has not been applied and embedded into their lives, including exploration of its most and least helpful components. We will also interview co-delivering clinicians and service team members (*n*=5) and supplement these with key informant interviews (service commissioners, policy-makers, national autism and third-sector leads; *n*=5) to give understanding of the broader organisational and systems contexts that may impact on intervention sustainability and roll out.

### Covid-19 pandemic modifications

Prior to the commencement of trial recruitment (Sept–Oct 2020), trial procedures were adapted in response to the pandemic and ongoing social distancing requirements in place at that time and which continued until at least 2022. These included online intervention delivery through a video-conferencing platform; data collection through remote procedures (video-conferencing, telephone, post, electronic correspondence); recorded verbal informed consent procedures; and the addition of a COVID impact questionnaire. All such adaptations were reviewed and approved by the Research Ethics Committee, funder, and sponsor, prior to implementation. In 2022, the target sample size was increased to maintain statistical power in response to early indicators of higher attrition than anticipated, attributed partly to pandemic-related factors; the timeline was extended accordingly. No further major modifications needed to be made to the design, participants, outcomes, randomisation or blinding procedures, or analyses (as of 2022) during the course of the trial.

## Methods: participants, interventions and outcomes

### Study setting {9}

Referral and treatment-as-usual sites are NHS paediatric, child and adolescent mental health and neurodevelopmental assessment services and local authority autism teams in the North West England, UK. Trial intervention groups take place online. A list of study sites is included in the Acknowledgments section.

### Eligibility criteria {10}

Eligibility for study centres: NHS teams that provide autism assessment and diagnosis (child development centres, community paediatric services, and child and adolescent mental health services) and local authority teams that provide services for families of autistic children (including those newly diagnosed). To be eligible, centres must (1) not already use the Manchester University NHS Foundation Trust post-diagnostic workshop approach from which the Empower-Autism programme was developed, to mitigate any contamination across trial arms; (2) be able to ring-fence any equivalent treatment-as-usual programme (to offer access to TAU participants and restrict access to intervention arm participants); and (3) make enough diagnoses per year to allow efficient recruitment to planned centre-specific clusters.

Eligibility for individuals who will perform the interventions:

- Lead practitioners: Trial-specific NHS Practitioners will be recruited to deliver the Empower-Autism programme, trained and supervised by Co-applicants Hackett and Dunkerley and an ACT Consultant. To be eligible, they will have the following: a recognised relevant clinical qualification and registration, good all-round knowledge of autism, minimum of 3 years’ experience working with families of autistic children, experience of delivering group-based intervention groups to caregivers, and skilled in both delivering didactic content and in facilitating group learning processes. They will be trained in acceptance and commitment therapy as part of their induction and will receive ongoing ACT-informed supervision. To remain eligible, they require ongoing delivery of intervention with satisfactory fidelity.
- Local co-delivering practitioners: Within each referral centre, local clinicians will work in collaboration with the Practitioners to offer session-specific expertise and localisation and to build capability and sustainability within local teams. For eligibility, they will require a professional role within the collaborating clinical team, good all-round understanding of autism and the autism context, and experience of running group-based interventions with parents/caregivers
- Eligibility for participants: All individuals will be considered for participation in this study regardless of age, disability, gender reassignment, marriage and civil partnership, pregnancy and maternity, race, religion and belief, sex, and sexual orientation except where the study inclusion and exclusion criteria explicitly state otherwise.

Inclusion criteria:

- At consent, child aged between 2 years 0 months and 15 years 11 months. This is the age-range typically seen by autism diagnostic teams.
- At referral, child given a diagnosis of ASD from an NHS professional within the last 12 months.
- One “index” adult per child (child’s parent/primary caregiver; must be aged 18 years or over), nominated by family on “intention to participate” basis.
- Child diagnosed with ASD is a patient/service user of one of the trial collaborating centres.

Exclusion criteria:

- Adult with insufficient English to preclude participation
- Adult with significant learning disability or significant hearing/visual impairment to preclude participation
- Adult with current severe psychiatric condition to preclude participation
- Significant current safeguarding concerns within family, identified by referring clinician

### Who will take informed consent? {26a}

Informed consent will be taken by University of Manchester researchers who have received mandatory training in Good Clinical Practice, which includes informed consent. Following the referral of a potential participant, a member of the research team will contact the individual and have a more detailed discussion about the trial, go through the Participant Information Sheet, and answer any questions they may have. Once the individual has had sufficient time to consider participating (minimum of 48 h), ask questions, and discuss it with family and friends, the researcher will proceed with fully informed consent. This will take place over video-conferencing by default, but over the telephone in the event of severe Internet connection issues, and will be audio-recorded.

### Additional consent provisions for collection and use of participant data and biological specimens {26b}

Additional optional consent is taken by researchers, following trial informed consent, for future contact in the event of a follow-up study or other related research studies.

### Interventions

#### Explanation for the choice of comparators {6b}

The trial tests the new Empower-Autism programme plus treatment-as-usual (TAU) against the usual local post-diagnostic offer plus TAU. The comparator is therefore any post-diagnostic, group-based, psycho-education intervention available as part of the standard local offer. Any such TAU intervention, identified in discussion with each trial centre, is offered as per standard practice to families randomised to the TAU arm, but is restricted to families randomised to the experimental intervention arm who access the Empower-Autism programme in its place. Participants in both trial arms can access all other services and interventions on offer in their locality, as per usual care.

With regard to the risk of contamination across trial arms, it is unlikely that families in the intervention arm will be close to other families in the TAU arm and unlikely that detailed intervention information would be shared between participants.

#### Intervention description {11a}

##### Treatment-as-usual

Standard care pathways are specified, for example, in the NICE guidelines, but vary considerably across services and NHS Trusts. Within the trial, participating families randomised to TAU will access their usual local post-diagnostic, group-based, autism psycho-education single- or multi-session programme offer (where one exists; in some localities there is no offer) plus any more general TAU services and interventions on offer in their locality, as per usual care (which may include, for example, individual review, individual needs-led interventions, needs-led group-based interventions, onward referral, etc). Centre differences in TAU will be captured via detailed service-use data collection and factored into the design and analyses by stratifying the randomisation by centre.

##### Experimental intervention

Participants randomised to the experimental treatment arm will access the Empower-Autism programme instead of their usual local post-diagnostic group-based programme offer (where one is offered). Like the TAU group, they will continue to access any general TAU services and interventions on offer in their locality, as per usual care. The Empower-Autism programme is a closed-group manualised and holistic programme, designed to address the needs of a wide range of caregivers in the post-diagnostic period, with a particular focus on caregiver mental health and wellbeing. It is composed of five 3-h sessions, delivered online via video-conferencing. Each programme is delivered by a lead and supporting practitioner and attended by a maximum of 12 participants (with one additional non-trial adult per family, if desired). The programme integrates:

- *Autism psycho-education*: The programme consists of high-quality, up-to-date, and evidence-informed autism psycho-educational content, including: information about autism, the diversity and complexity of autism presentations including reflection on how it presents within their own child; core characteristics of autism including communication, social interaction, thinking styles, sensory needs, emotion and energy regulation, and behaviour; and child-centred strategies to support each of these areas. First-hand autistic perspectives are included through videos, descriptions, quotes, and exercises and through signposting to materials that offer personal accounts. The aim of the psycho-educational content is caregiver understanding, insight, empathy, and empowerment through knowledge of the condition, practical strategies for use in daily life, and ability to make informed and positive choices for ongoing care. A range of delivery methods are employed to ensure sessions are enjoyable and interactive; to share information, ideas, and experiences; and to consolidate, apply, and extend learning.
- *Autism context*: This content comprises information about the education system as it relates to children and young people with special educational needs and disabilities, reflection on working together with educational settings, and sources of local, national, and online sources of information and support.
- *ACT content and philosophy*: Empower-Autism offers an experiential and skills-based introduction to ACT tools, philosophy, and processes to support participants with their own emotional responses to the diagnosis and parenting their child, and to adapt and flex to any challenges that may arise as a result of their child’s condition, the surrounding context, or any other factors. Emphasis is placed on the need for caregivers to value and give time and space for their own emotional wellbeing and stress management. The programme adopts the ACT Matrix [44] as the main delivery framework and includes the following concepts: mindful awareness and pausing; mindful noticing of thoughts, feelings, and physical sensations; cognitive defusion (choosing whether or not to engage with unhelpful thoughts and unhooking from them); expansion (accepting and making space for difficult thoughts and feelings rather than fighting against them); values clarification (reflecting on what is important to you as a person and as a caregiver); and committed action (making a commitment to small steps in a direction consistent with your values). These principles are introduced and reinforced through group discussion, experiential exercises, metaphors, videos, and individual reflection and tasks and are modelled by group facilitators within all discussions and activities. The ACT philosophy asserts the shared humanity and expertise of participants and group facilitators, autism- and neurodiversity-positivity, flexibility and responsiveness to individual needs and group dynamics, and acceptance of and compassion for the diverse range of thoughts, feelings, and experiences brought by group members.
- *Social support and validation* is promoted through group formation, networking, sharing of experiences and expertise, facilitated group discussions, and linkage to local, national, and online sources of community. Optional ongoing informal peer support is encouraged, for example, through a closed messaging or social media group.
- *Extension resources:* Extended learning related to the psycho-education and ACT components is offered through physical resources (handouts and information sheets) and online resources through a secure web portal developed by the team during the pre-trial phase and hosted on the University of Manchester server. This includes signposting to existing highly regarded local and national websites, organisations, and sources of information. Home practice suggestions are made at the end of each session and consist of exercises, strategies, or reflections that can be carried out as part of daily life.

#### Criteria for discontinuing or modifying allocated interventions {11b}

The trial intervention would be discontinued by the research team for a given trial participant if there was a serious adverse event and/or multiple adverse events, possibly or definitely related to the intervention. The intervention would also be discontinued at participant request or decision. Participants may withdraw from the trial or intervention programme at any time, without giving a reason. The research team may withdraw a participant for welfare or safeguarding reasons, or if participation is no longer in their interest. The research team will update a withdrawal log stating the date and reason for withdrawal. Data collected up until the point of withdrawal will be used. Participants will not be replaced.

#### Strategies to improve adherence to interventions {11c}

Intervention fidelity (practitioner adherence): Intervention variability will be minimised through frequent individual and group supervision of lead practitioners. During all sessions, the lead practitioners (but not participants) will be videotaped with audio recording of practitioners and participants. A random sample of 10% of sessions will be assessed formally for fidelity to the manual by a fully trained (including ACT-trained) independent senior practitioner. This will occur at regular intervals across the intervention period. To test the reliability of these ratings, 25% of fidelity sessions will be double-scored by a second rater, also a fully trained (including ACT-trained) senior practitioner. A core competency framework which assesses key psycho-education and ACT components and ACT processes will be used to formally rate fidelity and will be accessed throughout the study by the Practitioners to aid their self-assessment of adherence. Feedback from fidelity ratings will be shared with practitioners via their usual lines of supervision. Where fidelity drops to below threshold on the scale, remedial action will be taken. If ratings fall below this threshold on a consistent basis, the individual will discontinue intervention delivery.

Participant adherence: Research staff and intervention practitioners will be trained in practices that minimise non-compliance and drop-out. The expectation that participants attend the full course of five sessions is emphasised pre-randomisation by researchers and post-randomisation by intervention practitioners. Participant attendance at and engagement with the sessions will be documented following each session. In the event of missed sessions, practitioners will contact participants to explore the reasons for non-attendance and to offer a summary of the information covered within the session, including signposting to related information in the handouts and intervention online resources. Receipt of other interventions outside of the protocol will be collected.

#### Relevant concomitant care permitted or prohibited during the trial {11d}

Participants within the TAU arm will be permitted to access any concomitant care or interventions. Participants within the intervention arm will be permitted to access any concomitant care or intervention that does not constitute their usual local post-diagnostic, group-based, autism psycho-education single- or multi-session programme offer.

#### Provisions for post-trial care {30}

There are no provisions for post-trial care. The trial is low-risk and it is not anticipated that participants will suffer significant harm from trial participation; there is no provision for compensation to those who suffer harm from trial participation.

### Outcomes {12}

#### Primary outcome

Caregiver mental health, measured by the General Health Questionnaire-30 (GHQ-30; [45]), measured by the total score at baseline and 12-, 26-, and 52-week follow-up. The GHQ is the gold standard self-report measure of mental health in the general population or within community/non-psychiatric clinical settings. It is widely used in mental health trials, is well-validated, and has excellent sensitivity to change and psychometric properties, yielding normally distributed data [45]. There is no well-validated blinded measure of caregiver mental health for use within clinical trials. The GHQ is appropriate to measure mental health needs this context, in which caregivers are epidemiologically at risk but are not themselves selected on the basis of a mental health diagnosis. It will be, therefore, more sensitive to change than other screens/diagnostic tools designed for psychiatric populations. It provides a unitary measure of symptoms of both depression and anxiety. It assesses current mental state, rather than long-standing attributes of the respondent, and is therefore suited to measuring shorter term change that may be influenced by the child’s recent diagnosis. The GHQ has been successfully used to show a treatment effect on mental health in caregivers of autistic children in several previous studies [46], including an RCT of a psycho-education group [22] and an observational study of ACT [29]. The GHQ-30 is the most widely validated version of the GHQ with over 29 validity studies [45]. It was developed from the GHQ-60 but takes half the time to complete (3–4 min as opposed to 6–8 min)—important when participants will be completing several questionnaires at multiple time points. At an item level, the GHQ-30 is more appropriate than the GHQ-28 to administer to this non-clinical patient group (e.g. fewer items on suicidality). The GHQ-30 has published clinical cut-offs so rates of caseness can be used to assess meaningful and clinically significant change alongside the total score as a measure of absolute change.

#### Secondary outcomes

Caregiver measures, total scores measured at baseline and 12-, 26-, and 52-week follow-up (unless otherwise stated):

- Caregiver autism knowledge (Knowledge of Autism Questionnaire-UK), adapted from previous knowledge questionnaire for current UK context (administered at baseline and 12 and 52 weeks only)
- Caregiver wellbeing and quality of life, using the Warwick and Edinburgh Mental Wellbeing Scale [47]
- Caregiver Health Status, using the Euroqol EQ-5D-5L-Self reported version [48]
- Caregiver adjustment to diagnosis (The Reaction to Diagnosis Questionnaire; [49]) (administered at baseline and 52 weeks only)
- Parenting stress (Autism Parenting Stress Index; [50])
- Parenting self-efficacy (Tool to measure Parenting Self Efficacy; [51])
- Caregiver psychological flexibility (Acceptance and Action Questionnaire-II; [52])

*Family measures, measured at baseline and 52-week endpoint:*

- Family wellbeing, by a caregiver-nominated self-report measure of family experience and wellbeing developed through parent consultation within our previous trials (Autism Family Experience Questionnaire; [53])
- Expressed Emotion as a blind-rated measure of family emotional climate (Autism Five Minute Speech Sample; [54])

*Child measures at baseline and 52-week endpoint (unless otherwise stated):*

- Child adaptive functioning (caregiver-rated Vineland Adaptive Behaviour Scales; [55])
- Child wellbeing and health status (caregiver-rated Child Health Utility-9D Index; [56])
- Child emotional and behaviour difficulties (caregiver- and teacher (blind)-rated Strengths and Difficulties Questionnaire; [57])

### Sample characterisation measures

Collected at baseline: Demographics (including caregiver age and ethnicity, child age, family socio-economic status, number of people in the household, number and age of children cared for by the index caregiver, languages spoken); clinical information (date of child’s autism diagnosis, other child medical diagnoses, caregiver mental health or neurodevelopmental diagnoses; medical diagnoses of siblings); child autism severity (Social Communication Questionnaire; [58]); and adaptive behaviour [55] as a proxy for IQ.

Collected at 12 weeks: Caregiver measure of subthreshold autism traits (Subthreshold Autism Questionnaire; [59]). Collected at baseline and 12-, 26-, and 52-week follow-up: Impact of COVID-19 and the pandemic (COVID-19 Impact Questionnaire)

### Service use

A Health and Social Care Service-Use Interview (SUI) will be administered at baseline and 26- and 52-week follow-up. The SUI will include questions about whether the caregiver and child have used any primary, secondary, or community-based health and social care and how often they used the service in the last 6 months (baseline study visit) or since the last assessment (follow-up study visits). A separate form (Caregiver Group-based Interventions Questionnaire) will ask participants about autism-specific services group-based interventions accessed. Combining these forms with the SUI will provide a picture of the range of services used by caregivers and children in usual care. The SUI will also include whether the caregiver was absent from work due to their own ill health or their child’s ill health or care needs. The SUI will be developed from existing autism related SUIs held by the co-applicants and through discussion with the parent and public involvement representative, advisory group, and clinical members of the study team.

### Participant timeline {13}

**Fig. 1:**
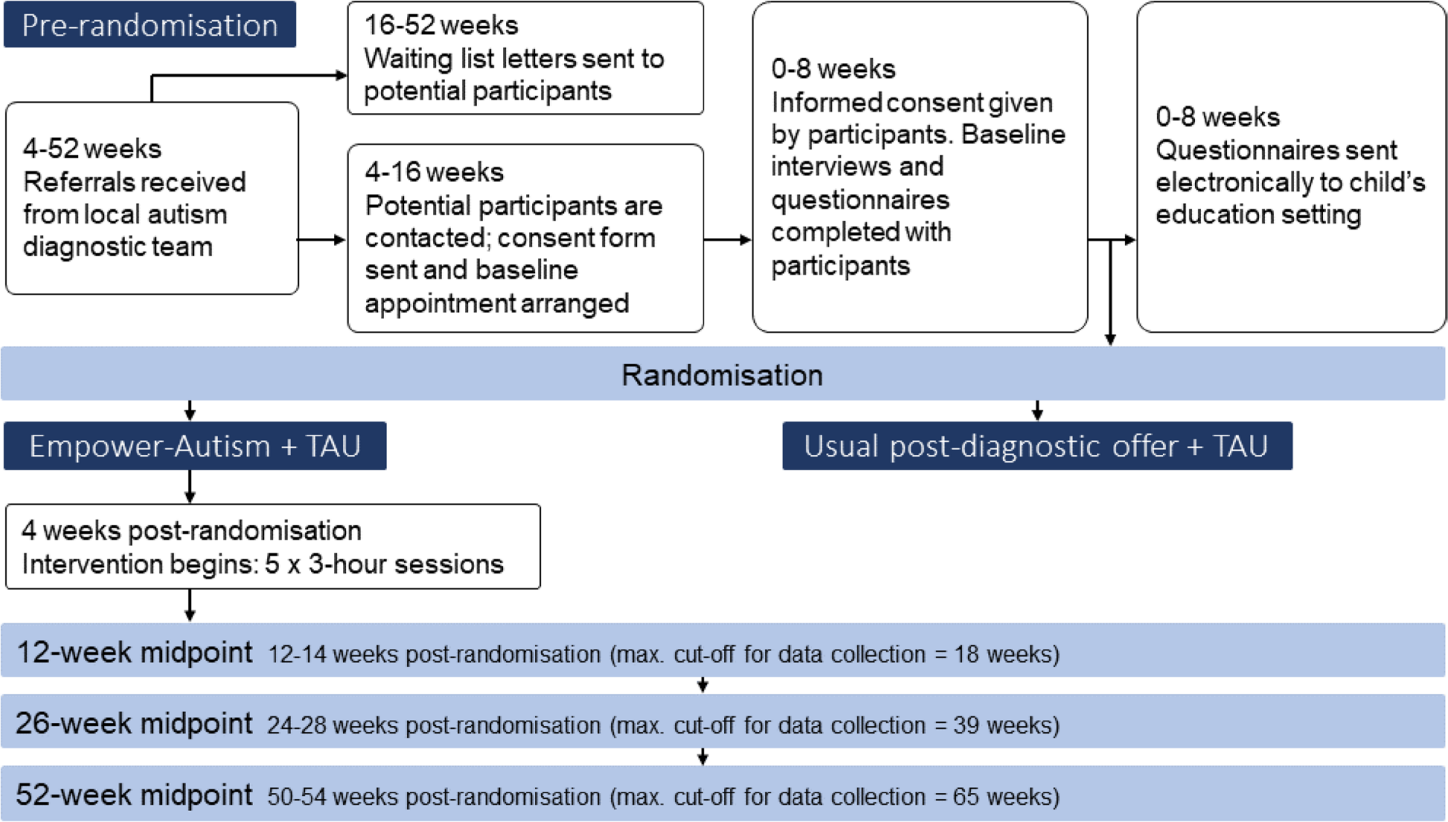
Flow of participants through the trial

The participant timeline is shown in Fig. 1.

### Study procedures by visit

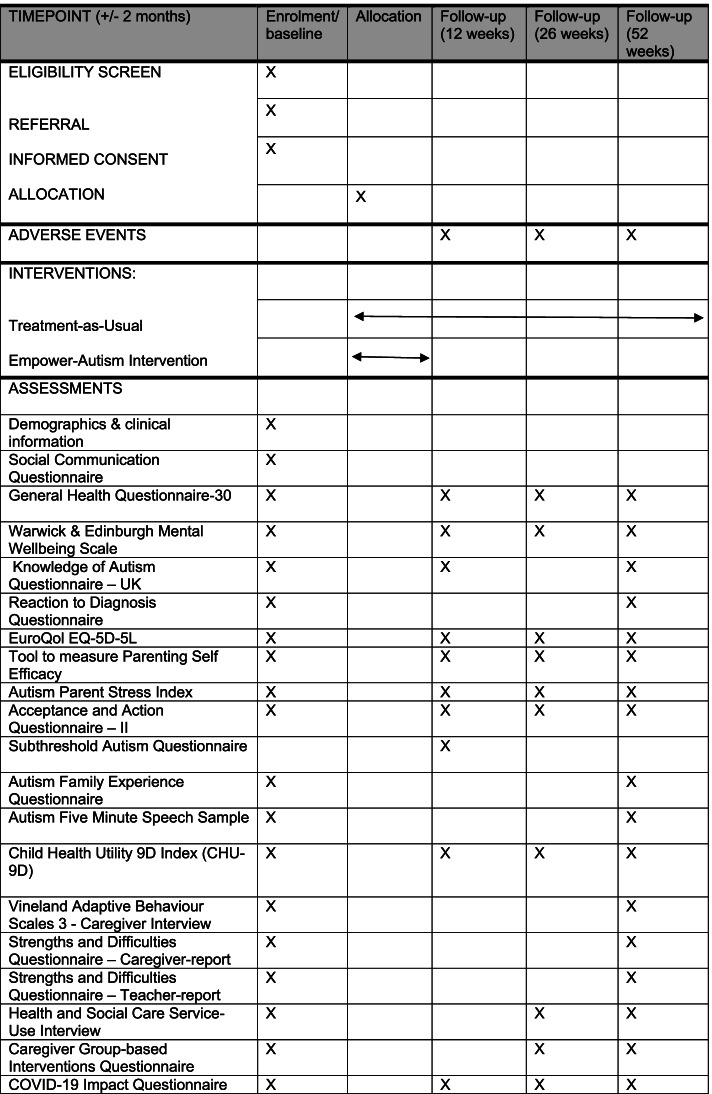



### Sample size {14}

#### Original sample size calculations

Using the Stata clsampsi command, we powered on the basis of minimum clinical superiority compared to TAU for an effect size of 0.4 based on effects in similar trial [38]. Nuisance parameters in the sample size calculations included as conservative estimates. We account for differential clustering because of the partial nested design, with groups of size 10, variation in group size of 10 and ICC=0.02 in treatment arm, and considering participants in TAU-only arm as clusters of size 1; baseline-endpoint correlation of 0.3; a two-sided significance level of 0.05; 2:1 allocation ratio. For 90% power, we require 285 participants in the analysis set: 190 participants in the treatment arm and 95 in TAU. An estimate of attrition of 15% across both arms gives a recruitment total of 330 participants; 22 groups of size 10 in the treatment arm. In a general adult population survey, the GHQ-30 had a standard deviation of 10.8; hence, a 0.4 effect size corresponds to a 4.3 point change [60].

#### Revised sample size calculations

At the request of the funder, the sample size calculation was re-performed in November 2021. Due to the probable effects of the COVID pandemic, attrition was higher than originally anticipated. A more conservative 25% attrition was used in the revised sample size and power calculations. These were based on groups of average size 8 and variation in group size of 8 with ICC=0.02 in treatment arm, and an increased attrition rate of 25%. 90% power requires 285 participants in the analysis set: 192 participants in the treatment arm and 93 in TAU. An estimate of attrition of 25% across both arms gives a recruitment total of 380 participants; 32 groups of average size 8 in the treatment arm (about 256 participants randomised to treatment overall) and about 124 participants randomised to the TAU-only arm.

### Recruitment {15}

Multiple strategies will be used to achieve adequate participant enrolment to reach the target sample size. We will build upon excellent pre-existing relationships with local autism teams through our previous trials and the clinical work of co-investigators and benefitting from the integrated service development and commissioning of the Greater Manchester Health and Social Care Strategic Partnership. We will prioritise collaborating with referring diagnostic teams that assess large numbers of children and will open up new referral sites, as necessary, to access new participant waiting lists. Potential participants will be initially approached by a clinician from their local team, where possible, to maximise initial trust and engagement with the trial. Within the trial itself, there will be flexible scheduling of research appointments to accommodate participants’ preferences. Intervention groups will also run on a range of days and in daytime and evening to enable participation from caregivers with professional and caring commitments. The internal pilot recruitment phase will provide an initial test of recruitment potential, with pre-specified progression criteria based on satisfactory recruitment (using the same rate as will be needed to achieve full recruitment within the wider trial).

## Assignment of interventions: allocation

### Sequence generation {16a}

Participants will be randomly allocated to one of two trial arms using a bespoke web-based randomisation system developed and managed by the King’s Clinical Trials Unit (KCTU) which is hosted on a dedicated server within Kings College London. Batch individual randomisation will be by child, with one “index” caregiver per child, and stratified by centre, using a 2:1 randomisation ratio to assist recruitment and deliver timely intervention with 2 participants being allocated to the intervention arm for every 1 participant allocated to the TAU arm.

### Concealment mechanism {16b}

As randomisation is performed in batches through an independent service at KCTU, the allocation sequence will be concealed until participants are assigned allocations.

### Implementation {16c}

Participants will be enrolled and consented into the trial by University researchers. Baseline assessments will be undertaken prior to treatment assignment. Participants are assigned by the researcher/Trial Manager to one particular randomisation batch. The default is for consented participants to be placed into the next randomisation batch corresponding to the centre from which they were referred. However, there is flexibility to be randomised within a different batch if this is a better fit with individual participant availability to attend intervention sessions (if randomised to the intervention arm). The list of participants for each batch will be sent through to the KCTU who will carry out batch randomisation. Unblinded treatment allocation emails will be sent to the Trial Manager informing which group each participant in that batch was assigned to. The Trial Manager then informs the NHS Practitioners the allocation of each participant in the randomisation batch. NHS Practitioners then contact each family via email/letter and phone call informing them of their allocation. Participants allocated to the intervention group will be invited to the sessions, and those allocated to treatment-as-usual will be linked back into local provision.

## Assignment of interventions: blinding

### Who will be blinded {17a}

All data collection staff and their supervisors will be kept blind to group allocation; intervention practitioners and supervisors and families cannot be blinded. Caregiver-rated primary and secondary outcomes are not blind-rated; researcher-scored/coded secondary outcomes will be blinded (and subject to reliability checking), as will teacher-rated secondary child outcomes. We have established blinding procedures from our previous trials. There will be separate clinical and research leads and separate training and supervision structures. Researchers will be housed separately from staff involved in training and delivery of the Empower-Autism intervention. Mid- and endpoint research assessments will be conducted to avoid inadvertent divulging of information that could infer treatment status. Data collection staff will be uninformed on the details of the intervention. The senior statistician will be kept blind throughout the trial; the junior statistician will become unblinded once the statistical analysis plan (SAP) is signed off prior to accessing any outcome data that is required to be summarised split by arm, as this will unblind due to unequal arm allocation. All analysis will be pre-specified in the SAP and the trial dataset will be generated with a dummy variable for group allocation so that the primary analysis code can be reviewed by the blinded senior statistician.

### Procedure for unblinding if needed {17b}

Individual researchers may be unblinded to a participant’s allocation if necessary in the event of a serious adverse event.

## Data collection and management

### Plans for assessment and collection of outcomes {18a}

Recruitment of participants will be via our partner NHS/local authority diagnostic teams. A member of the local clinical team will initially identify potential participants and screen for eligibility. An NHS practitioner/clinician will then provide a brief introduction of the trial and, with consent, pass over the contact details to the University research team.

All research activity and data collection will be carried out by trained, blinded Research Associates/Assistants in accordance with pre-specified trial standard operating procedures. Due to the COVID-19 pandemic, research activities will take place remotely. Following a referral, an introductory email and the Participant Information Sheet will be emailed to the potential participant. A member of the research team will then contact the family and have a more detailed discussion about the trial, go through the Participant Information Sheet with the participant and answer any questions they may have. Once participants have had sufficient time to consider participating, ask questions, and discuss it with family and friends, the researcher will proceed with fully informed audio-recorded verbal consent. Each case will be registered and assigned a participant ID number. The baseline assessment will then be undertaken by Research Assistants prior to randomisation. Follow-up data collection will take place at 12, 26, and 52 weeks after randomisation.

Data collection will take place remotely wherever possible. Questionnaire data will be collected via post or electronically via email, online survey or via telephone and/or videoconferencing, with visits to participants’ homes only when necessary and COVID-related guidelines permit. Researchers will sit and/or discuss over the phone/videoconference with participants during questionnaire completion to assist with understanding where necessary and to minimise missing data. Interviews will also be completed remotely via telephone and/or videoconferencing, or at the participant home. The Autism Five Minute Speech Sample will be audio-recorded, transcribed, and coded by research staff. Teacher questionnaires will be collected at baseline and endpoint either via school/nursery visits and/or remotely. Data collection forms are available through their publishers/authors; trial-specific forms are available on request.

Process evaluation data will be collected post-endpoint for a subgroup of participants, sampled purposively, via remote (telephone or videoconferencing) semi-structured interviews. There will be an additional Participant Information Sheet and Consent Form for this data collection.

### Plans to promote participant retention and complete follow-up {18b}

To promote retention and complete follow-up data collection, the timing and methods of follow-up appointments will be discussed with participants at baseline and an appointment card and customised fridge magnet will be sent to participants. A £40 thank-you voucher is given to participating caregivers at completion of the endpoint questionnaire; school staff are sent a £10 voucher to acknowledge their efforts in questionnaire completion. Regular trial newsletters will be sent to participating families, thanking them for their commitment to the trial.

### Data management {19}

Data management procedures can be found in a Standard Operating Procedure, available on request from the corresponding author. All data in the trial will be anonymised. A central master file of personal data will be held securely in the University of Manchester research office, to be used for operational purposes, and this will contain the key linking anonymised participant IDs to personal details. Trial data will be entered by research staff into the online Elsevier MACRO EDC system, developed and hosted by the KCTU with double data checking for 100% of primary outcome, the first two cases by each individual conducting data entry, and for a random 10% of cases. The trial database has monitoring functions and a full audit trail. Appropriate quality control will be carried out during the trial and before the database lock.

Data protection will be specified and followed, in keeping with Good Clinical Practice and the General Data Protection Regulation 2018. All personal data and audio and video recordings will be held on password protected University of Manchester and NHS secure servers. Copies may also be held in a locked cabinet, on encrypted hard-drives in accordance with pre-specified highly secure procedures. All data will be kept confidential, accessed only by the trial team.

Data will be stored in the Faculty of Biology, Medicine and Health, University of Manchester. Paper copies will be stored centrally in secured cabinets. Electronic data will be stored within the Kings College Clinical Trials Unit secure data storage facility and on University of Manchester supported research storage systems. At the end of the trial, the data will be stored for a period of 15 years before being destroyed. The data custodian will be Professor Jonathan Green, Chief Investigator of the study.

### Confidentiality {27}

Confidentiality procedures will be pre-specified and followed, in keeping with Good Clinical Practice and the General Data Protection Regulation 2018. Personal information will be collected by researchers during research data collection. Personal information may be shared outside of the research team only with participant consent, e.g. with clinicians involved with the family. The only time that personal information will be shared without consent is if there are serious concerns about the safety or wellbeing of a child or vulnerable adult. In this event, local procedures for safeguarding children and vulnerable adults will be followed. Data collection forms will be identifiable only by participant ID and will contain no names or contact details. Personal and sensitive data will be stored separately and securely on a password-protected section of the university/NHS servers and/or hard drive in secure offices located at University of Manchester and NHS facilities. If personal information needs to be emailed, this will be in an encrypted form.

### Plans for collection, laboratory evaluation, and storage of biological specimens for genetic or molecular analysis in this trial/future use {33}

Not applicable; no biological specimens will be collected.

## Statistical methods

### Statistical methods for primary and secondary outcomes {20a}

A detailed statistical analysis plan will be approved by the Data Monitoring and Ethics Committee (DMEC) and Trial Steering Committee before analysis of unblinded data. Analysis will follow intention-to-treat principles and follow the CONSORT statement for non-pharmacological interventions. Analyses will post-date final follow-up assessments, with due consideration of potential biases from loss to follow-up. Baseline data will be presented using appropriate summary statistics with no testing for baseline differences.

To satisfy Objective 1, treatment effects on the primary and secondary clinical outcomes will be estimated using linear mixed models fitted to outcome variables at all time points. Fixed effects will be centre, baseline assessment for the outcome under investigation, treatment, time, and time×treatment interactions. Participant and group number will be included as random intercepts, treating the control participants as ‘groups’ of size 1. Marginal treatment effects will be estimated for outcomes at each timepoint, and reported separately as mean adjusted differences in scores between the randomised groups with 95% confidence intervals and two-sided *p*-values. The random effect structure will account for repeated measures and clustering due to the partial nested design and allow estimates of the ICC in the intervention arm.

For secondary outcomes only measured at baseline and 52 weeks, the same approach will be used without the time×treatment interaction and time as fixed effects, since there is only one measurement occasion.

For all analyses, each intervention group will contain only the outcome measures on an index caregiver, and so beyond the group-level clustering, no further adjustment for multiple caregivers is required.

#### Health economic analysis

The economic analysis will use a within-trial, intent to treat approach and include all participants randomised to the two trial arms. The primary analysis will use the NHS and social care (costs) and parents/primary caregivers (health benefits) perspectives, with a 12-month time horizon. Costs will be estimated from health and social care service use data collected via the SUI and Caregiver Group-based Interventions Questionnaire. National unit costs will be used to cost each of the services used [61, 62]. Health benefit for the primary analysis is the QALY (EQ-5D-5L version, and published utility tariffs recommended by NICE at the time of the analysis). Health economic regression analysis, adjusted for key covariates, will estimate the net costs and QALYs of the intervention. Missing data will be accounted for in the analyses of net costs, net QALYs, and cost effectiveness acceptability. The methods used to deal with missing follow-up data will be determined according to the extent and pattern of missing data [63–65]. The estimates of net costs and QALYs from the regression analyses will be bootstrapped [66] to simulate 10,000 pairs of incremental cost and QALY outcomes of the intervention. This will include (i) plotting the distribution of pairs of net costs and QALYs on a cost-effectiveness plane, (ii) generating a cost-effectiveness acceptability curve, (iii) estimating the probability that intervention is cost-effective, and (iv) estimating a net benefit statistic. Sensitivity analyses will explore the intervention’s cost-effectiveness using (i) GHQ-30 (caregiver mental health), (ii) the adapted Child Health Utility-9D Index (child wellbeing), and (iii) wider perspective to include indirect costs of lost productivity. A simple decision model will explore the potential cost-effectiveness of the intervention over longer time horizons.

A detailed economics analysis plan will be approved by the DMEC and TSC prior to analysis. The economic evaluation will be reported in line with the Consolidated Health Economic Evaluation Reporting Standards (CHEERS) statement [67].

### Interim analyses {21b}

There will be no planned interim analysis for efficacy or futility.

### Methods for additional analyses (e.g. subgroup analyses) {20b}

No subgroup analyses are planned.

### Methods in analysis to handle protocol non-adherence and any statistical methods to handle missing data {20c}

The primary analysis will be on the intention-to-treat population using a linear mixed model. This approach will allow for missing outcome data under the Missing At Random assumption; we may also use inverse probability weighting to adjust for non-adherence to allocated treatment and other intermediate outcomes as predictors of future loss to follow-up.

### Plans to give access to the full protocol, participant-level data, and statistical code {31c}

The datasets generated and analysed and the corresponding statistical code will be available in anonymised form from the research team on reasonable request, subject to review, following the publication of trial results.

## Oversight and monitoring

### Composition of the coordinating centre and trial steering committee {5d}

The project management group is responsible for the day-to-day conduct of the trial and will meet regularly, at least quarterly, across the course of the trial. The group will be chaired by the Chief Investigator and will comprise trial principal investigators, statisticians and health economists, trial manager, and members of the research and NHS intervention teams, and other invited members as necessary.

The Trial Steering Committee (TSC) will be formed, including an independent chair, parent representatives, an NHS clinician, and an experienced triallist (TSC members listed in the Acknowledgments). The TSC will be consulted on the design, protocol, techniques for ascertainment, and measurement. The TSC will meet at least once prior to the commencement of the trial and at least annually thereafter.

### Composition of the data monitoring committee, its role and reporting structure {21a}

There will be an independent data monitoring and ethics committee (DMEC) comprising three external members who bring methodological, statistical, clinical, and subject-specific expertise and declare no conflicts of interest (DMEC members listed in the Acknowledgements). Its role will be to safeguard the interests of trial participants, assess the safety of the interventions during the trial, and monitor overall trial conduct. The DMEC is independent of the sponsor and funder and will report to the Trial Steering Committee. The DMEC Charter is available on request from Prof Richard Emsley, King’s College London.

### Adverse event reporting and harms {22}

For all participants, we will collect information about adverse events at each follow-up visit and record adverse events in a standard format. We will capture adverse events that pertain to the trial index adult and the index child. In addition to recording medical adverse events in the standard way, we will also include events particularly relevant to this psycho-social intervention/trial, such as those relating to wellbeing, mental health, and difficult family circumstances.

Information about the adverse event will be collected, including but not exclusive to categorisation, description of event, length of time, start/stop date, ongoing status, relationship to intervention, and whether it is deemed a serious adverse event. It is possible that during the intervention sessions, participants may report “adverse events” to the Trial Practitioners. These will be recorded as part of the therapy log that practitioners will complete after each session. These events will be referred to as ‘therapy reported negative events’ as they will only be applicable to one arm of the Trial. These events will be monitored internally as there is the risk of unblinding researchers. Any serious events will however be reported the same as adverse events.

Adverse events will be monitored by the DMEC and TSC. Serious adverse events will be reported to the project management group and sponsor. If any of the serious adverse events are a suspected unexpected reaction to the intervention (it is acknowledged that this is highly unlikely in this trial), these will be reported immediately to the sponsor, research ethics committee and DMEC.

### Frequency and plans for auditing trial conduct {23}

Trial conduct will be monitored by regular auditing visits from the sponsor, annual reports to the NHS Research Ethics Committee, and bi-annual reports to the funder.

### Plans for communicating important protocol amendments to relevant parties (e.g. trial participants, ethical committees) {25}

Protocol amendments will be communicated to all relevant parties, including the Project Management Group, trial sponsor, Health Research Authority and Research Ethics Committee, funder, the oversight committees, and the trial registry (ISRCTN). Trial participants will be informed where it is relevant to their participation.

## Dissemination plans {31a}

The trial results will be submitted for publication in peer-reviewed journals of general and special interest. There will also be a general dissemination programme for families co-ordinated through autism@manchester, Autistica, NHS England, Greater Manchester Health and Social Care Partnership, and the National Autistic Society. Feedback for participating clinical teams and participants will be shared through the regular trial newsletter. Authorship on dissemination papers will follow International Committee of Medical Journal Editors guidelines and journal requirements. There will be no use of professional writers.

## Discussion

Care pathways for autistic children and their families need to be proactive, evidence-based, developmentally phased, and scalable [7]. This trial addresses a key evidence gap required within such a care pathway model by providing a robust and well-powered test of a new intervention programme designed to be inclusive and deliverable within a publicly funded health system and to meet the diverse informational, emotional, and relational needs of caregivers in the weeks and months following their child’s diagnosis. The provision of careful, targeted, and holistic support at this critical time will potentially improve wellbeing and prevent escalation of need for both caregivers and their autistic children. If shown to be clinically and cost effective, this programme will fill an important evidence gap within current care pathways within the UK and internationally.

The trial adopts an ambitious primary outcome: caregiver mental health across a 12-month period, with a range a secondary outcomes relating to caregiver and family wellbeing, understanding, and functioning. Blind-rated outcomes complement those that measure first-hand experience. The trial will therefore generate knowledge about the impact of the Empower-Autism programme on diverse facets of caregiver experience, but also any downstream effects on the family and autistic child. Health economic analysis will provide important information about the costs and benefits of this comprehensive programme, compared to standard caregiver post-diagnostic psycho-education offers. Importantly, the nested process evaluation will inform implementation and sustainability beyond the trial.

The trial commenced in October 2020 coinciding with the first year of the COVID-19 pandemic; significant national and local restrictions were in place throughout 2020–2022. Modifications to remote intervention and research delivery were made prior to the commencement of the trial and were then applied throughout the trial timeline. The pandemic brought significant practical and emotional challenges to families of children with disabilities within the UK [68] and these challenges will impact upon trial recruitment and retention. To mitigate this, the following measures have been taken: increase in sample size to mitigate attrition, addition of COVID impact questionnaire to measure the adverse effects of the pandemic on individual families across the timeline of their participation in the trial, and qualitative data on effects of pandemic collected within the process evaluation.

## Trial status

Protocol version number: 8 dated 24/02/2022

Date recruitment began: 21/09/20

Recruitment was completed on: 19/04/22

## Supplementary Information

**Additional file 1.**

## Data Availability

Primary analysis of the data will take place by the trial statisticians. Other team members will have access to data and will be able to undertake analysis as appropriate and in accordance with a publication protocol agreement.

## Abbreviations

ACT: Acceptance and commitment therapy
ASD: Autism spectrum disorder
DMEC: Data Monitoring and Ethics Committee
GHQ: General Health Questionnaire
KCTU: Kings College Clinical Trials Unit
NDD: Neurodevelopmental disabilities
NHS: The United Kingdom National Health Service
NICE: National Institute for Health and Care Excellence
NIHR: National Institute for Health and Care Research
PSSRU: Personal Social Services Research Unit
QALY: Quality-adjusted life years
RCT: Randomised controlled trial
SAP: Statistical analysis plan
SUI: Health and Social Care Service-Use Interview
TAU: Treatment-as-usual
TSC: Trial Steering Committee
UK: United Kingdom
