# Supplementary material for: REACH-ASD: a UK randomised controlled trial of a new post-diagnostic psycho-education and acceptance and commitment therapy programme against treatment-as-usual for improving the mental health and adjustment of caregivers of children recently diagnosed with autism spectrum disorder
Source: Trials. 2022 Jul 22;23:585. doi: 10.1186/s13063-022-06524-1 (PMC9306249; doi:10.1186/s13063-022-06524-1)

**
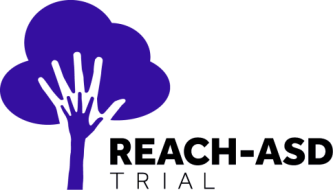
**

Participant ID: ________________

**REACH-ASD Trial: A Randomised Controlled Trial of Psycho-Education and Acceptance & Commitment Therapy for Parents of Children recently diagnosed with ASD**

**MAIN TRIAL CONSENT FORM ***

Please initial box **

1. I confirm that I have read the information sheet dated current date (current version) for the above study. I have had the opportunity to consider the information, ask questions and have had these answered satisfactorily.
2. I understand that my participation is voluntary and that I am free to withdraw at any time
   without giving any reason, without my health care or legal rights being affected.
3. I agree to my child’s education setting being contacted so a key member of staff can complete

questionnaires about my child.

4. I understand that I will be randomly allocated to one of two groups: my usual local post-diagnostic offer OR the EMPOWER-ASD programme. I will do my best to attend the group I am allocated to. I understand I am able to access any other available services or therapies that I would usually receive

1. I understand that the information collected about me will be used to support other research in

the future, and may be shared anonymously with other researchers.

1. I agree to audio-recording being used in this study, with possible use of anonymised quotations.

I understand that all audio-recordings will be stored securely and used for research purposes only.

1. I agree to video-recording being used in this study. I understand that all video-recordings will

be stored securely and used for research purposes only.

1. I agree to my General Practitioner being informed of my participation in the study.
2. I agree to take part in the REACH-ASD study.

_____________________________ ________________ ___________________________

Name of Participant Date Signature

________________________________ ________________ ___________________________

Name of Researcher Date Signature

*In the event of verbal consent, a recording of confirmation will be made available to the participant.

**Verbal agreement by the participant to each statement read by the researcher.

1 copy is to be given to the participant; 1 copy to be stored at the research site

Version no: v2 Date: 24.06.2020 IRAS No: 268914


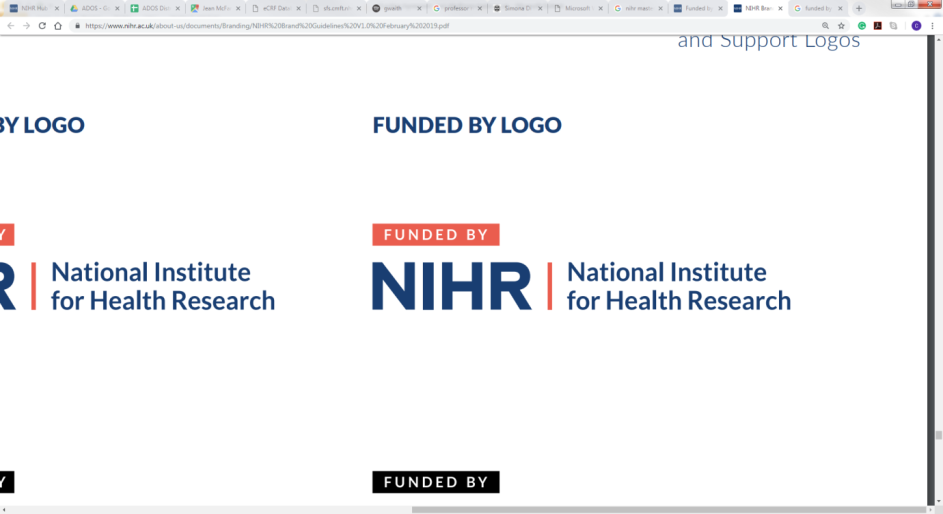

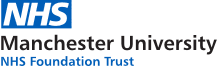

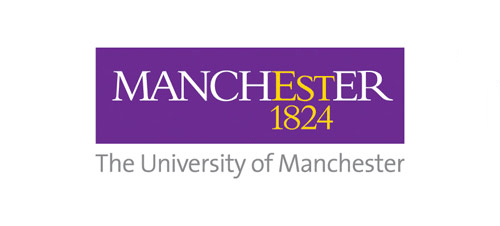

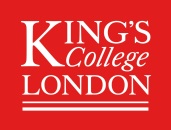

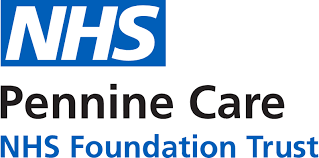

Supplement: Supplementary file 1 — Additional file 1. [file 13063_2022_6524_MOESM1_ESM.docx]
